# Functional Relationship of Arabidopsis AOXs and PTOX Revealed via Transgenic Analysis

**DOI:** 10.3389/fpls.2021.692847

**Published:** 2021-07-02

**Authors:** Danfeng Wang, Chunyu Wang, Cai Li, Haifeng Song, Jing Qin, Han Chang, Weihan Fu, Yuhua Wang, Fei Wang, Beibei Li, Yaqi Hao, Min Xu, Aigen Fu

**Affiliations:** ^1^Chinese Education Ministry’s Key Laboratory of Western Resources and Modern Biotechnology, Key Laboratory of Biotechnology Shaanxi Province, College of Life Sciences, Northwest University, Xi’an, China; ^2^College of Life Sciences, Northeast Agricultural University, Harbin, China

**Keywords:** alternative oxidase (AOX), plastid terminal oxidase (PTOX), mitochondria, chloroplasts, targeting peptide, protein dual targeting

## Abstract

Alternative oxidase (AOX) and plastid terminal oxidase (PTOX) are terminal oxidases of electron transfer in mitochondria and chloroplasts, respectively. Here, taking advantage of the variegation phenotype of the Arabidopsis PTOX deficient mutant (*im*), we examined the functional relationship between PTOX and its five distantly related homologs (AOX1a, 1b, 1c, 1d, and AOX2). When engineered into chloroplasts, AOX1b, 1c, 1d, and AOX2 rescued the *im* defect, while AOX1a partially suppressed the mutant phenotype, indicating that AOXs could function as PQH_2_ oxidases. When the full length AOXs were overexpressed in *im*, only AOX1b and AOX2 rescued its variegation phenotype. *In vivo* fluorescence analysis of GFP-tagged AOXs and subcellular fractionation assays showed that AOX1b and AOX2 could partially enter chloroplasts while AOX1c and AOX1d were exclusively present in mitochondria. Surprisingly, the subcellular fractionation, but not the fluorescence analysis of GFP-tagged AOX1a, revealed that a small portion of AOX1a could sort into chloroplasts. We further fused and expressed the targeting peptides of AOXs with the mature form of PTOX in *im* individually; and found that targeting peptides of AOX1a, AOX1b, and AOX2, but not that of AOX1c or AOX1d, could direct PTOX into chloroplasts. It demonstrated that chloroplast-localized AOXs, but not mitochondria-localized AOXs, can functionally compensate for the PTOX deficiency in chloroplasts, providing a direct evidence for the functional relevance of AOX and PTOX, shedding light on the interaction between mitochondria and chloroplasts and the complex mechanisms of protein dual targeting in plant cells.

## Introduction

Plant cells harbor two energy-converting organelles, mitochondria and chloroplasts, which evolved from ancient prokaryotes through different endosymbiotic events (Cavalier-Smith, 2000; Leister, 2003). Most of the symbiont genes were lost or transferred to their host genomes during evolution. Expression of the remaining small genomes in organelles and of the nuclear genome of host cells is highly coordinated (Martin and Hermann, 1998; Abdallah et al., 2000). Mitochondria and chloroplasts are tightly linked to each other, sharing some common conserved proteins (Mueller and Reski, 2014). Photosynthesis provides carbon substrates to sustain mitochondrial respiration, and mitochondria contribute essential metabolites to maintain chloroplast functions as well (Blanco et al., 2014; Zhao et al., 2018).

The respiratory electron transfer chain (RETC) in plant mitochondria consists of a main cytochrome pathway and an alternative pathway (Møller, 2001; Finnegan et al., 2004; Schertl and Braun, 2014). Electron transfer in the cyanide-sensitive cytochrome pathway is coupled to transmembrane proton translocation and responsible for ATP synthesis (Juszczuk and Rychter, 2003). The alternative pathway, mediated by a cyanide-resistant alternative oxidase (AOX), catalyzes electron transfer from UQH_2_ (ubiquinol) to oxygen (Vanlerberghe and Mclntosh, 1997; Juszczuk and Rychter, 2003). This energy-wasteful alternative pathway oxidizes reducing equivalents without coupling to proton translocation across the mitochondrial membrane and ATP synthesis (Rogov et al., 2014; Vishwakarma et al., 2015).

The photosynthetic electron transfer chain (PETC) in chloroplasts consists of photosystem II, PQ (plastoquinone), cytochrome b_6_f complex, plastocyanin and photosystem I (Rochaix, 2011; Hasan et al., 2013). In prokaryotic cyanobacteria, PETC and RETC coexist in the thylakoid membrane and share some intermediate components (Myers, 1986; Cooley et al., 2000). As the evolutionary remnants of cyanobacteria, chloroplasts also contain an O_2_-dependent electron transfer pathway, referred to as chlororespiration, which involves the NADH dehydrogenase complex, the PQ pool, and the plastid terminal oxidase (PTOX) (Bennoun, 2002). As a distant homolog of mitochondrial AOX (Carol et al., 1999; Wu et al., 1999), PTOX transfers electrons from PQH_2_ to oxygen at the thylakoid membrane, mimicking the function of AOX at the mitochondrial inner membrane (Nawrocki et al., 2015).

PTOX and AOX are derived from an ancient di-iron oxidase, but they diverged early on different evolutionary routes before the endosymbiotic events giving rise to mitochondria and chloroplasts (McDonald and Vanlerberghe, 2006; Nobre et al., 2016). AOX is a UQH_2_ oxidase in mitochondria, while PTOX is a PQH_2_ oxidase in chloroplasts. *In vitro* enzyme assays showed that PTOX specifically uses PQH_2_ as substrate and AOX exclusively uses UQH_2_ (Josse et al., 2003; Fu et al., 2012; Yu et al., 2014).

The PTOX-mediated electron transfer pathway does not contribute significantly in chloroplasts when PETC develops and functions well (Fu et al., 2009; Nawrocki et al., 2015). However, it might be a major force to drive electron transfer in darkness, in non-photosynthetic plastids, or at the early stage of chloroplast development (Wang and Fu, 2016). PTOX is a key factor for maintaining the PQ pool redox balance and functions as a “safety valve” to protect photosynthesis (Wang and Fu, 2016). It is a stress-responsive protein and could protect plants from various harmful stresses (Laureau et al., 2013; Stepien and Johnson, 2018). PTOX is also critical for carotenoid biosynthesis since inactivation of PTOX results in a deficiency of phytoene desaturation, a key step in carotenoid biosynthesis (Okegawa et al., 2010; McDonald et al., 2011). Consequently, the Arabidopsis PTOX null mutant, *immutans* (*im)*, shows a striking light-dependent variegation phenotype due to the absence of protective carotenoids (Wu et al., 1999; Fu et al., 2005, 2009, 2012). In addition, PTOX participates in chloroplast development and plant photomorphogenesis (Foudree et al., 2012; Tamiru et al., 2013).

As a non-energy conserving terminal oxidase, AOX keeps a balance between mitochondrial carbon and energy metabolism. Similar to PTOX, AOX also plays a critical role in response to different types of stress conditions, including low/high temperature (Murakami and Toriyama, 2008; Wang et al., 2011), salinity (Wang et al., 2010), metal toxicity (Tan et al., 2010), high light/drought (Zhang et al., 2010, 2012; Yoshida et al., 2011a), high CO_2_ (Gandin et al., 2012), low oxygen (Clifton et al., 2005), nutrient deficiency (Juszczuk et al., 2001), and bacterial infection (Spoal and Loake, 2011).

There are broad metabolite exchanges and signal communications between mitochondria and chloroplasts, and an orchestrated coordination of these two organelles is essential for plant cells (Selinski and Scheibe, 2019). Recent data showed that AOX plays a central role in coordinating signaling pathways between mitochondria and chloroplasts (Vanlerberghe et al., 2020). Analysis of plant *aox1a* mutants illustrated that AOX could protect photosynthesis from photo-damage by dissipating excess reducing power in chloroplasts (Yoshida et al., 2007, 2008, 2011b; Zhang et al., 2010, 2016). It is generally accepted that the malate/oxaloacetate (Mal/OAA) shuttle connecting chloroplasts and mitochondria is responsible for transferring excess reducing equivalents from chloroplasts to mitochondria (Noguchi and Yoshida, 2008; Taniguchi and Miyake, 2012; Zhang et al., 2012; Vishwakarma et al., 2015). Recently, photorespiration was also found to be involved in the AOX-mediated protection of photosynthesis (Li et al., 2020).

Besides providing a mitochondrial means to indirectly optimize the chloroplasts energy status, AOX could directly enter chloroplasts and regulate PETC in the thylakoid membrane (Fu et al., 2012). Overexpressing AOX2 in Arabidopsis *im* rescued the variegation phenotype of the mutant, and AOX2 was found dually targeted to mitochondria and chloroplasts. In addition, when engineered into chloroplasts, AOX1a could partially suppress the growth defect of *im* (Fu et al., 2012). These results demonstrated that both AOX2 and AOX1a could act as PQH_2_ oxidase in chloroplasts, suggesting that the substrate specificity of PTOX and AOX in plants may not be as stringent as shown by *in vitro* enzyme assays (Fu et al., 2012).

Targeting peptide analysis indicated that the presence of multiple arginine and hydrophobic residues in the N-terminal region would enable a protein to be preferentially imported into mitochondria rather than into chloroplasts (Ge et al., 2014; Lee et al., 2019). All five Arabidopsis AOX members (AOX1a-1d and AOX2) fit the profile of mitochondria specifically targeted proteins, and PTOX also matches the characteristic of chloroplast targeted proteins. However, the fact that AOX2 is a dually targeted protein suggested that AOX proteins could be imported into chloroplasts although they are predominantly present in mitochondria (Fu et al., 2012).

Taking advantage of the striking variegation phenotype of the Arabidopsis *im* mutant, we explored the functional relevance of AOXs and PTOX by overexpressing various forms of AOXs in *im*. Following the previous study which revealed AOX2 is a dually targeted protein and chloroplast-localized AOX1a could partially rescue *im* (Fu et al., 2012), this study attempts to answer the following questions: whether all AOXs could act as PQH_2_ oxidase, whether elevated levels of AOXs attenuate the deficiency of electron transfer in *im*, and whether they are dually targeted proteins to mitochondria and chloroplasts. We found that all five AOXs could utilize PQH_2_ as a substrate in chloroplasts, and AOX1a, AOX1b, and AOX2 could be dually targeted proteins. We concluded that AOXs could enter chloroplasts to regulate electron transfer in chloroplasts whereas mitochondria-localized AOXs do not play a significant role in compensating the PTOX deficiency in chloroplasts. This study suggested that the cellular compartmentation of proteins in plant cells might not be as strict as previously assumed, and there could be a certain plasticity for proteins to sort into different subcellular locations.

## Materials and Methods

### Primers

All the primers used in this study are listed in Supplementary Table 2.

### Plant Materials and Growth Conditions

The plants in this study included the wild type, *im*, and transgenic plants with the genetic background of *Arabidopsis thaliana* Columbia-0. Because *im* is light-sensitive, the plants were germinated and grown under low light (∼20 μmol⋅m^–2^s^–1^) for the initial 5 days, and then transferred to normal light (∼100 μmol⋅m^–2^s^–1^). All plants in this study were grown on soil at 23°C with a photoperiod of 16 h light/8 h dark cycle.

### Constructing Transgenes and Plant Gene Transformation

Five Arabidopsis *AOX* CDS (*AOX1a*, *AOX1b*, *AOX1c*, *AOX1d*, and *AOX2*) were obtained by RT-PCR using primers listed in Supplementary Table 2, respectively.

For chloroplast expression, the coding sequence of five AOX mature proteins (full length AOX minus the mitochondrial targeting peptide) were fused to the Arabidopsis RbcS1A chloroplast targeting peptide (CTP) driven by the CaMV 35S promoter (P35S) in the binary vector pB003 (Fu et al., 2012). The resulting plasmids were respectively designated as C-mAOX1a, C-mAOX1b, C-mAOX1c, C-mAOX1d, and C-mAOX2.

For overexpressing the full length AOX proteins with their own targeting sequences, the five *AOX* CDS were fused to P35S in pB003, and the five resulting plasmids were named as W-AOX1a, W-AOX1b, W-AOX1c, W-AOX1d, and W-AOX2, respectively.

A cDNA fragment encoding the mature form of Arabidopsis PTOX (the coding sequence of PTOX minus its own targeting peptide) was generated by RT-PCR. The amplified sequence was fused behind the coding sequences for the five AOX targeting peptide (TP) in pB003, respectively; the resulting plasmids were designated as AOX1aTP-mPTOX, AOX1bTP-mPTOX, AOX1cTP-mPTOX, AOX1dTP-mPTOX, and AOX2TP-mPTOX. The PTOX mature protein sequence was cloned into pB003 as control, in which only the initiation codon ATG was added in front of mature PTOX resulting in the plasmid ATG-mPTOX.

All constructs were confirmed by sequencing, then transferred into *Agrobacterium tumefaciens* strain GV3101 and further introduced into *im* by the floral dip method (Clough and Bent, 1998). BASTA-resistant T1 transgenic plants were selected on soil by spraying 2-week-old seedlings with 150 mg/L Basta solution (Sangon Biotech, China), and verified by PCR.

### RNA Extraction and RT-PCR Analysis

Total RNA was extracted from 3-week-old Arabidopsis leaves using the RNAPrep pure plant kit (Tiangen, China). RNA was reversely transcribed to the 1st strand cDNA using the PrimeScript II 1st strand cDNA Synthesis Kit (Takara, Japan). All the experiments above were performed according to the manufacturer’s protocol. *ACTIN2* gene was used as control. PCR were performed with an initial pre-denaturation of 98°C 30 s, followed by 28 cycles of 98°C for 15 s, 55°C for 30 s, and 72°C for 1 min.

### Subcellular Localization by Fluorescence Analysis of GFP-Tagged Proteins

To determine the subcellular location of AOXs with the Arabidopsis RbcS1A CTP, the five AOX mature CDS were respectively cloned behind the Arabidopsis RbcS1A CTP and inserted into pSPY-GFP (Zhang et al., 2018). The five fusion constructs were designated as C-mAOX1a-GFP, C-mAOX1b-GFP, C-mAOX1c-GFP, C-mAOX1d-GFP, and C-mAOX2-GFP, respectively.

To determine the subcellular location of AOXs with their own targeting peptides, the GFP sequence was inserted behind the AOX CDS in W-AOX1a, W-AOX1b, W-AOX1c, W-AOX1d, and W-AOX2; the resulting constructs were designated as W-AOX1a-GFP, W-AOX1b-GFP, W-AOX1c-GFP, W-AOX1d-GFP, and W-AOX2-GFP, respectively.

To determine the subcellular location of PTOX with different AOX targeting peptides, the five fragment of the AOX1aTP-mPTOX, AOX1bTP-mPTOX, AOX1cTP-mPTOX, AOX1dTP-mPTOX, and AOX2TP-mPTOX were respectively inserted before the GFP sequence in the plasmid pCAMBIA3300. The resulting constructs were designated as AOX1aTP-mPTOX-GFP, AOX1bTP-mPTOX-GFP, AOX1cTP-mPTOX-GFP, AOX1dTP-mPTOX-GFP, and AOX2TP-mPTOX-GFP.

All above constructs were introduced into the *Agrobacterium tumefaciens* GV3101 strain, and co-infiltrated with the p19 strain into *N. benthamiana* leaves for transient expression as described in Fu et al., 2012. The transfected plants were incubated at 23°C for 48∼72h before taking images. Bright-field and fluorescent microscopy were performed on an Olympus Fluoview FV1000 confocal laser scanning microscope (Olympus, Japan). The fluorescent images were taken with excitation at 488 nm and emission at 500–530 nm for detection of GFP. Mt-Mcherry were excited using 543 nm and detected in red detection channels of 570–625 nm. The chloroplasts autofluorescence were excited at 594 nm and measured with an emission at 625–725 nm. *pGreen-35S:Mt-Mcherry* was used as mitochondrial marker.

### Antibody Generation

Polyclonal antibodies were generated as described previously with minor modifications (Chen et al., 2000). Five AOX polyclonal antibodies were raised in rabbits against the specific and soluble coding region of each AOX expressed in *Escherichia coli* using the pGEX-4T.3 vector system (Takara, Japan). These proteins were purified by Glutathione Sepharose 4B (GE Healthcare, United States) affinity chromatography. Rabbits were immunized by serial injections of the purified specific and solubilized protein. Then, the serum were collected from immunized rabbits. Immune complexes were detected by the eECL Western Blot Kit (CWBIO, China) using a secondary antibody conjugated to horseradish peroxidase (Bioworld, United States). The chemiluminescent signals were captured with a CCD camera (Tanon 5200, China). The specific antigen peptide sequence of each AOX is listed in detail as follows:

AOX1a:ASTITLGEKTPMKEEDANQKKTENESTGGDAA GGNNKGDKGIA;
AOX1c: SKMTFEKKKTSEEEEGSGDGVKVNDQGNKG EQLIV;
AOX1d: LSSDTSSPVSGNNQPENPIRTADGKVISTYW GIP;
AOX2: GMSSASAMEKKDENLTVKKGQNGGGSVAVPSY WGIETA.

### Preparation of Total Leaf Proteins, Intact Chloroplast Proteins, and Immunoblotting Analysis

Total leaf proteins were extracted by grinding leaf material and resuspended in 2x SDS loading buffer [125 mM Tris-HCl, pH 6.8, 4% (w/v) SDS, 2% (v/v) 2-mercaptoethanol, 0.001% (w/v) bromophenol blue, 20% (v/v) glycerol], followed by incubation at 98°C for 5 min, centrifugation at 13,800 *g* for 10 min.

Intact chloroplasts were isolated using the Percoll gradient method (Perry et al., 1991; Bhuiyan et al., 2015). The 4-week-old Arabidopsis leaves were ground in a blender with ice-cold homogenization buffer (20 mM Tricine-KOH, pH 8.4, 450 mM sorbitol, 10 mM EDTA, pH 8.0, 10 mM NaHCO_3_, and 0.1% BSA). Homogenates were then filtered through two layers of Miracloth. The filtrate was the total cell proteins, and crude chloroplasts were collected by centrifugation at 1,000 g for 10 min at 4°C. Resuspended crude chloroplasts in 1 mL of resuspension buffer (20 mM Tricine-KOH, pH 8.4, 300 mM sorbitol, 2.5 mM EDTA, and 5 mM MgCl_2_) were gently overlaid on Percoll step gradients (40 and 80% v/v), and were centrifuged at 4,000 *g* for 20 min at 4°C. Intact chloroplasts were collected from the interface between the 40 and 80% Percoll layers with burned tips and washed once with resuspension buffer.

Immunoblotting analysis were conducted as described (Fu et al., 2012). Briefly, protein samples were electrophoresed through 12% SDS polyacrylamide gels and transferred to nitrocellulose membranes for immunoblotting analysis. The protein signals were visualized using the eECL Western Blot Kit (CWBIO, China) and captured with a CCD camera (Tanon 5200, China).

### *In vivo* Chlorophyll Fluorescence Measurements

Chlorophyll fluorescence measurements were performed with Dual-PAM-100 fluorometer (Heinz-Walz, Germany) on intact leaves from wild-type (WT), *im*, W-AOX1b, and W-AOX2 transgenic plants grown for 7 weeks in soil. F_v_’/F_m_’ was measured by rapid light curves. During this process, the leaves were irradiated for 30 s under different light intensities. F_v_’/F_m_’ was calculated as (F_m_’-F_*o*_’)/F_m_’; ΦPSII was equal to (F_m_’-F)/F_m_’; 1-qP was defined as (F_*o*_’/F)(F_m_’-F)/(F_m_’-F_*o*_’); NPQ was calculated as (F_m_-F_m_’)/F_m_’. F_m_ is the maximum fluorescence in the dark-adapted state; F_m_’ is the maximum fluorescence in any light-adapted state; F_v_’ is the variable fluorescence equal to (F_m_’-F_o_’); F is the steady state fluorescence in the light; F_o_’ is the minimal fluorescence in any light-adapted state.

NPQ induction and dark kinetics measurements were performed as described previously (Fu et al., 2012). Plants were irradiated with actinic light of 1805 μmol m^–2^s^–1^ for 10 min following dark adaption. Relaxation data were acquired by shutting off the actinic source for an additional 5 min. During the entire time period, the fluorescence signals was superimposed with saturating 800-ms pulses of white light every 30 s.

### Isolation of Thylakoid Membrane and Blue Native Gel Electrophoresis

Thylakoid membranes were isolated as described previously with minor modifications (Lima et al., 2006). For blue native gel analysis, leaves were ground with a blender in ice-cold grinding buffer and filtered through two-layers of Miracloth. The filtrate was centrifuged at 2,000 *g* for 5 min at 4°C, the pellet was resuspended in SH buffer (330 mM sorbitol, 50 mM HEPES, pH 8.0). Thylakoid membranes were isolated by centrifugation at 2,400 *g* for 2 min at 4°C. Isolated thylakoid membranes were resuspended in 50BT40G buffer (50 mM BisTris, pH 7.0, and 40% glycerol) to a final chlorophyll concentration of 1 mg mL^–1^, and then solubilized with 1% (w/v) *n*-dodecyl-β-D-maltoside (Sigma-Aldrich, United States) for 5 min on ice. After centrifugation at 10,000 *g* for 10 min, the supernatant was mixed with 1/10 volume of BN sample buffer [100 mM BisTris-HCl, pH 7.0, 5% Serva blue G, 0.5 M 6-amino-*n*-caproic acid, 30% (w/v) sucrose]. Samples were loaded on a blue native gradient gel containing 5–13.5% polyacrylamide (Amresco, United States). Gel electrophoresis was performed at a constant voltage of 100 V for 6 h at 4°C.

### Sequence Alignment and Phylogenetic Tree

AOXs and PTOX mature protein sequences in Arabidopsis were compared in multiple sequence alignment using ClustalW program of MEGA6. The Neighbor-Joining method was used to build a phylogenetic tree with the bootstrapping value set at 1,000 replicates (Felsenstein, 1985; Saitou and Nei, 1987; Tamura et al., 2013).

### Accession Numbers

The sequence data for the following Arabidopsis genes can be found in the TAIR database^1^: AT3G22370 (*AOX1a*), At3G22360 (*AOX1b*), AT3G27620 (*AOX1c*), At1g32350 (*AOX1d*), At5G64210 (*AOX2*), and At4G22260 (*PTOX*), AT3G18780 (*ACTIN2*), AT1G12770 (*ISE1*).

## Results

### AOXs Substitute for the Function of PTOX After Targeting Into Chloroplasts

The Arabidopsis genome contains one PTOX (At4g22260) and five AOX members, including AOX1a (At3g22370), AOX1b (At3g22360), AOX1c (At3g27620), AOX1d (At1g32350), and AOX2 (At5g64210) (Saisho et al., 1997; Wang and Fu, 2016). Sequence alignment and phylogenetic analysis showed that Arabidopsis PTOX and AOXs are distantly related with 26% amino acid sequence identity (Supplementary Figure 1). Within the AOX family, AOX1a-1d are more closely related to each other, while AOX2 is distantly related to the AOX1 subfamily members. Among the four AOX1 members, AOX1b and AOX1c are the most closely related pair and evolved from a recent gene duplication event.

Although AOXs are UQH_2_ oxidases and PTOX is a PQH_2_ oxidase *in vitro* (Wang and Fu, 2016), AOXs could be PQH_2_ oxidases *in planta*, which was illustrated by the fact that the chloroplast-localized AOX1a and AOX2 acted as PQH_2_ oxidases in Arabidopsis (Fu et al., 2012). Here we further examined whether the other AOXs could function as PQH_2_ oxidases in chloroplasts.

**FIGURE 1:**
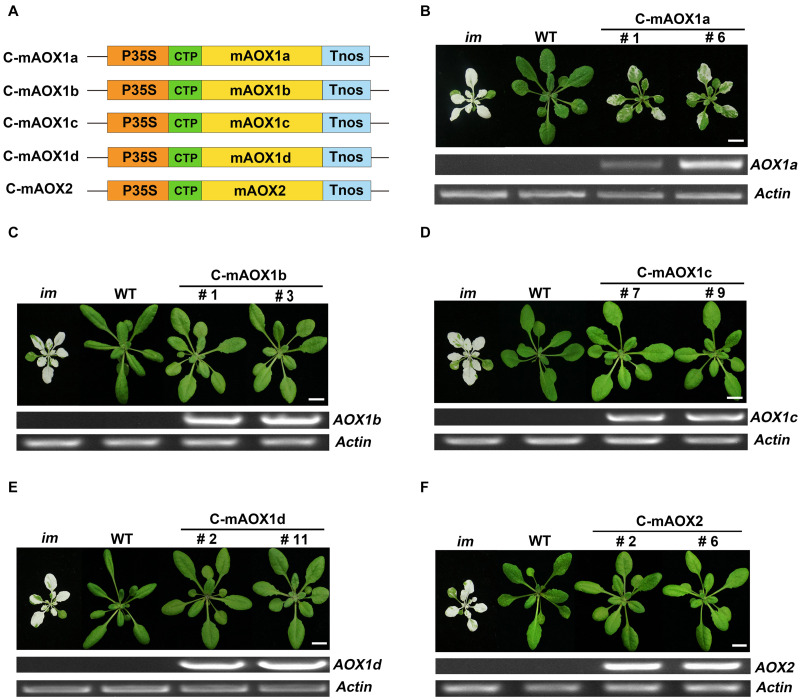
Expression of C-mAOX constructs in *im*. **(A)** Schematic diagram of C-mAOX1a, C-mAOX1b, C-mAOX1c, C-mAOX1d, and C-mAOX2. P35S, Cauliflower Mosaic Virus 35S promoter; Tnos, terminator of nos (nopaline synthetase); CTP, chloroplast targeting sequence from *Arabidopsis thaliana* RbcS1A (Rubisco small subunit); mAOX1a, mAOX1b, mAOX1c, mAOX1d, and mAOX2 are the mature proteins (the full length AOX minus the targeting peptide). **(B–F)** Phenotypes of *im* mutant, WT, and transgenic *im* mutants carrying 35S-driven C-mAOX1a, C-mAOX1b, C-mAOX1c, C-mAOX1d, and C-mAOX2. Expression of C-mAOX in *im* was examined at the RNA level by RT-PCR. All plants were grown for 4 weeks with a photoperiod of 16 h light/8 h dark cycle under light (∼100 μmol⋅m^–2^s^–1^) after initial low light (∼20 μmol⋅m^–2^s^–1^) growth for 5 days as described in the “Materials and Methods” section. Actin was used as quantity control. Bars = 1 cm.

We generated constructs containing AOX coding sequences with their targeting peptides replaced by the chloroplast-specific transit peptide of RbcS1A. The resulting constructs were referred as C-mAOX1a, C-mAOX1b, C-mAOX1c, C-mAOX1d, and C-mAOX2, respectively (Figure 1A). Then we introduced these constructs into the PTOX deficient mutant (*im*), and analyzed the phenotypes of transgenic plants of the T_2_ generation (Figure 1). Consistent with the previous study (Fu et al., 2012), none of the transgenic *im* plants bearing C-mAOX1a (more than 50 independent transgenic lines) showed a fully recovered WT like green phenotype, but leaf variegation was much less severe compared to *im* (Figure 1B). Transgenic *im* plants with the other C-mAOXs did not exhibit any variegation phenotype, and grew normally like WT plants (Figures 1C–F). We performed RT-PCR to examine the RNA level of AOXs in these transgenic plants and found that all transgenes are expressed well while the endogenous transcripts of all 5 AOXs are barely detectable in *im* and WT (Figure 1).

**FIGURE 2:**
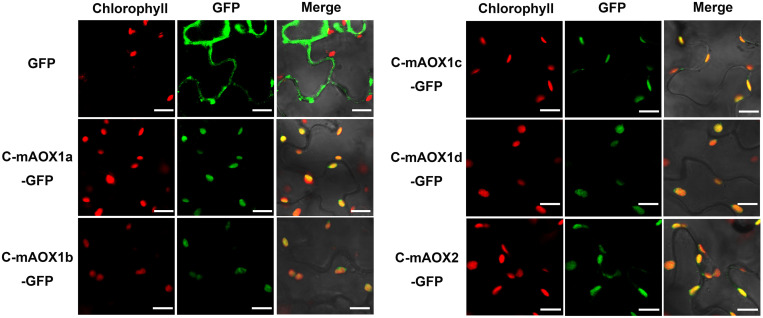
Subcellular localization of C-mAOX1a, C-mAOX1b, C-mAOX1c, C-mAOX1d, and C-mAOX2. C-mAOX1a, C-mAOX1b, C-mAOX1c, C-mAOX1d, and C-mAOX2 tagged with a C-terminal GFP were transiently expressed under the control of 35S promoter in *N. benthamiana* leaves and observed by confocal microscopy. In each case, images of chlorophyll autofluorescence (Chl), GFP fluorescence (GFP), and merged chlorophyll and GFP fluorescence with bright-field (Merge) are shown. Scale bar = 20 μm.

To examine whether the engineered C-mAOX proteins were successfully imported into chloroplasts under the guidance of the RbcS1A targeting peptide, we transiently expressed these recombinant C-mAOXs fused with GFP at their C-termini in *N. benthamiana*. Confocal images showed that all five C-mAOXs are targeted into chloroplasts, as illustrated by the overlay of the green fluorescence signals of GFP- tagged C-mAOXs with chlorophyll auto-fluorescence of chloroplasts (Figure 2).

Previous studies suggested that the ability to complement *im* was used as a proxy for the relative *in vivo* PQH_2_ oxidase activity in chloroplasts (Fu et al., 2005, 2009, 2012). Taken together, we concluded that all 5 chloroplast-localized AOXs could act as PQH_2_ oxidases and substitute for the function of PTOX to various degrees. Moreover, the PQH_2_ oxidase activity of AOX1a may be weaker than those of the other four homologs *in planta* and hence not able to fully complement the defect in *im* plastids. AOX1a has presumably been optimized for UQH_2_ as a substrate in mitochondrial membranes.

### Overexpression of the Full Length AOX1b or AOX2 in *im* Complements Its Variegation Phenotype

It is well accepted that AOXs are mitochondrial proteins. RNAseq analysis showed that AOXs are expressed at relatively low levels. In detail, *AOX1a* has a slightly lower expression level (about 15 RPKM) compared to *PTOX*, and the other four *AOX* genes are barely expressed in wild type plants (Supplementary Figure 2). Mitochondrial AOXs could dissipate excess reducing power in chloroplasts and protect photosynthesis from photo-damage through the Mal/OAA shuttle (Yoshida et al., 2011b; Zhang et al., 2016). Therefore, it is possible that overexpression of AOX might reduce the variegation phenotype of *im* through the crosstalk between mitochondria and chloroplasts. The other possibility is that overexpressed AOX could overflow from mitochondria and enter chloroplasts. This scenario was previously demonstrated that when overexpression of AOX2 in *im* enabled it to enter chloroplasts and replace the function of PTOX in Arabidopsis (Fu et al., 2012).

**FIGURE 3:**
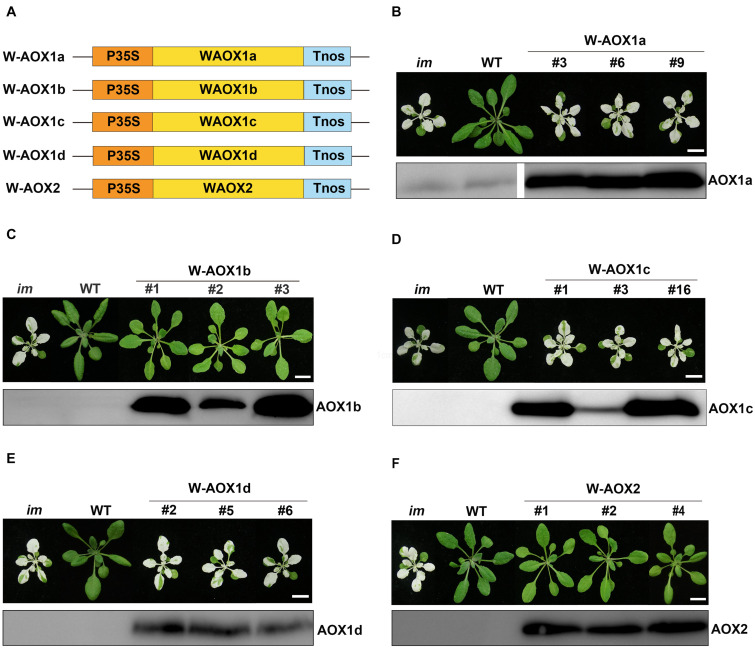
Expression of W-AOX constructs in *im*. **(A)** Schematic diagram of W-AOX1a, W-AOX1b, W-AOX1c, W-AOX1d, and W-AOX2 constructs. P35S, Cauliflower Mosaic Virus 35S promoter; Tnos, terminator of nos (nopaline synthetase); WAOX1a, WAOX1b, WAOX1c, WAOX1d, and WAOX2 are the full length coding sequences. **(B–F)** Phenotypes of *im* mutant, WT, and three independent lines of transgenic *im* mutants carrying 35S-driven W-AOX1a, W-AOX1b, W-AOX1c, W-AOX1d, and W-AOX2. All plants were grown for 4 weeks with a photoperiod of 16 h light/8 h dark cycle under light (∼100 μmol⋅m^–2^s^–1^) after initial low light (∼20 μmol⋅m^–2^s^–1^) growth for 5 days as described in the “Materials and Methods” section. Total cell proteins was isolated from 10 mg of fresh leaf tissue, and subjected to 12% SDS-PAGE and probed with the corresponding AOX antibodies. Bars = 1 cm.

To examine these possibilities, we overexpressed the full length AOXs in *im*, and performed analysis on T2 generation transgenic plants (Figure 3). More specifically, *im* plants were transformed with the full-length coding sequences of all five *AOXs* driven by the CaMV 35S promoter (named as W-AOXs, standing for the whole sequence of AOXs, in contrast to the mature AOX sequences tested in Figures 1, 2), separately (Figure 3A). In agreement with the previous study (Fu et al., 2012), transgenic *im* plants bearing the W-AOX2 construct produced green leaves similar to WT, distinct from the striking variegation phenotype of *im* (Figure 3F). Besides W-AOX2 plants, transgenic *im* plants with W-AOX1b also showed a green leaf phenotype similar to WT (Figure 3C), indicating that AOX1b could substitute for the function of PTOX as well as AOX2. On the other hand, transgenic *im* plants of W-AOX1a, W-AOX1c, and W-AOX1d displayed a typical variegation phenotype the same as *im* (Figures 3B,D,E), suggesting that overexpression of W-AOX1a, W-AOX1c, or W-AOX1d failed to complement the function of PTOX. This revealed remarkable differences among AOX proteins with respect to replacing the function of PTOX when they are overexpressed in *im*.

To test whether the distinct phenotypes of transgenic plants with different W-AOX constructs could be attributed to inefficiency in gene expression, we carried out immunoblotting analysis on transgenic plants with the respective AOX antibodies. Four polyclonal antibodies against AOX proteins (AOX1a, AOX1c, AOX1d, and AOX2) were raised against a specific peptide of each AOX, which was obtained from recombinant expression in *E. coli*. However, we failed to obtain a suitable antibody against AOX1b due to the difficulty to express a specific peptide of AOX1b in *E. coli*. Fortunately, the AOX1c antibody could detect AOX1b well, because of the high sequence identity (81%) between AOX1b and AOX1c. Immunoblotting assays showed that each AOX was overexpressed well in the corresponding transgenic *im* plants, in which much more proteins were accumulated than that in non-transgenic *im* or WT (Figure 3), indicating that the phenotypic differences of transgenic plants do not result from different protein levels. Notably, a trace amount of AOX1a was observed in *im* and WT, while the other four AOX proteins were barely detected in *im* and WT indicating low expression levels of AOX proteins in Arabidopsis under normal growth conditions (Figure 3).

### W-AOX1b and W-AOX2 Are Targeted Into Chloroplasts as Revealed by the Fluorescence Analysis of GFP-Tagged AOXs

**FIGURE 4:**
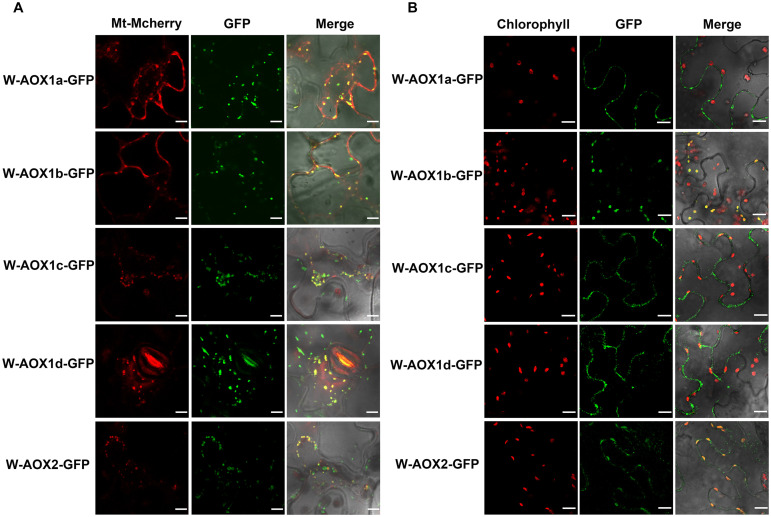
Subcellular localization of W-AOX1a, W-AOX1b, W-AOX1c, W-AOX1d, and W-AOX2. W-AOX1a, W-AOX1b, W-AOX1c, W-AOX1d, and W-AOX2 tagged with a C-terminal GFP were transiently expressed under the control of 35S promoter in *N. benthamiana* leaves and observed by confocal microscopy. **(A)** In each case, images of mitochondrial Mcherry fluorescence (Mt-Mcherry), GFP fluorescence (GFP), and merged Mcherry and GFP fluorescence with bright-field (Merge) are shown. Scale bar = 5 μm. **(B)** In each case, images of chlorophyll autofluorescence (Chl), GFP fluorescence (GFP), and merged chlorophyll and GFP fluorescence with bright-field (Merge) are shown. Scale bar = 20 μm.

All five AOXs are predicted to be present in mitochondria by various targeting prediction programs (Supplementary Table 1), and they could function as PQH_2_ oxidases when targeted to chloroplasts (Figure 1). While overexpression of W-AOX1b and W-AOX2 in *im* could rescue the mutant variegation phenotype, this was not observed when W-AOX1a, W-AOX1c, or W-AOX1d was overexpressed in *im* (Figure 3). This prompted us to consider that AOX1a, AOX1c, and AOX1d should be exclusively localized in mitochondria whereas AOX1b could be a mitochondria and chloroplasts dually targeted protein as AOX2. To examine this possibility, we tagged all five full-length AOXs (W-AOXs) with GFP at their C-termini, and transiently expressed these fusion proteins in *N. benthamiana.* Fluorescence image analysis showed that all five AOXs are mostly located in mitochondria as indicated by the fact that the green fluorescence signals of GFP-tagged W-AOXs overlaid well with the red fluorescence signals of Mt-Mcherry, the specific mitochondrial marker (Figure 4A). In contrast, several green fluorescence signals from GFP-tagged W-AOX1b and W-AOX2 evidently overlapped with the auto-fluorescence signals of chloroplasts (Figure 4B), indicating that, in addition to AOX2, AOX1b is also dually targeted to mitochondria and chloroplasts. With W-AOX1a, W-AOX1c, and W-AOX1d, we did not observe signals from the GFP-tagged proteins matching the auto-fluorescence signals of chloroplasts, indicating that these three AOXs are exclusively or predominantly localized in mitochondria.

### W-AOX1a Is Also Targeted to Chloroplasts as Shown by Immunoblotting

**FIGURE 5:**
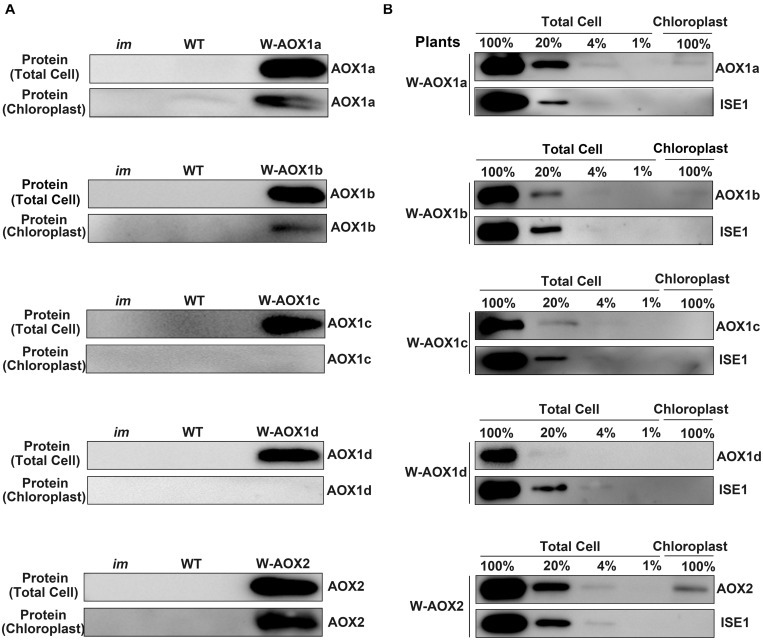
W-AOX1a, W-AOX1b, and W-AOX2 are present in chloroplasts of the overexpression lines. **(A)** Immunoblotting analyses. Rosette leaves from 4-week-old plants as in Figure 3 served as the source of total cell proteins and of chloroplast membranes from lysed, Percoll gradient-purified chloroplasts (designated chloroplasts). Equal protein amounts were electrophoresed through 12% SDS polyacrylamide gels, and immunoblotting analyses were performed with the corresponding AOX antibodies. **(B)** Total cell proteins or proteins from gradient purified chloroplasts were isolated from the rosette leaves of 4-week-old W-AOX1a, W-AOX1b, W-AOX1c, W-AOX1d, and W-AOX2 plants. Samples containing equal chlorophyll amounts (2 μg chlorophyll) were electrophoresed through 12% SDS polyacrylamide gels, and immunoblotting analyses were performed with antibodies against the corresponding AOX, and ISE1, a highly expressed mitochondrial-specific protein (Stonebloom et al., 2009). The gels contained a dilution series (100, 20, 4, and 1%) of total cell proteins.

To further verify the localization of AOXs in the transgenic *im* plants overexpressing W-AOXs, we purified chloroplasts by the Percoll gradient method and examined the distribution of AOXs in isolated chloroplasts by immunoblotting (Figure 5). The analysis showed that all five AOXs are barely detectable with their respective antibodies in total cell extracts or in purified chloroplast samples from *im* or WT plants (Figure 5A). However, a remarkably high level of each AOX was observed in the total cell samples from their respective transgenic *im* plants (Figure 5A). As expected, a substantial amount of AOX1b and AOX2 was observed in the chloroplast samples from W-AOX1b and W-AOX2 transgenic plants (Figure 5A) consistent with the plant phenotypes and the fluorescence analysis of GFP-tagged W-AOX1b and W-AOX2 (Figures 3, 4). In contrast, no detectable AOX protein was observed in the chloroplast samples from transgenic *im* plants overexpressing W-AOX1c or W-AOX1d (Figure 5A), also in agreement with the phenotypes of transgenic plants and the fluorescence analysis of GFP-tagged W-AOX1c and W-AOX1d (Figures 3, 4). To our surprise, a substantial amount of AOX1a was observed in the purified chloroplast samples from transgenic *im* plants overexpressing W-AOX1a (Figure 5A), which is in striking contrast to the variegation phenotype in the W-AOX1a transgenic *im* plants (Figure 3B) and the fluorescence analysis of GFP-tagged W-AOX1a (Figure 4B).

Given the high accumulation of AOX1a in transgenic *im* plants overexpressing W-AOX1a, it is possible that the detected signals of AOX1a in chloroplasts may arise from the contamination of mitochondrial samples. In order to examine the extent of mitochondrial contamination, we included a mitochondrial specific RNA helicase, ISE1 (Stonebloom et al., 2009), in the immunoblotting titration experiments (Figure 5B). The result showed that the ISE1 signals were remarkably strong in the total cell samples of all five transgenic plants, but not detectable in their chloroplast samples, demonstrating that the purified chloroplasts were barely contaminated with mitochondria (Figure 5B). The immunoblotting assays showed that AOX1c and AOX1d are absent in the chloroplast fractions, again confirming that they are mitochondrial specific proteins. The content of AOX1a, AOX1b, and AOX2 in isolated chloroplasts was much lower than that in the total cell samples, suggesting that the majority of the overexpressed AOX1a, AOX1b, and AOX2 are not present in chloroplasts, and should be normally in mitochondria. Based on the titration assays, we estimated that approximate 10% of AOX2, 4% of AOX1a and AOX1b can be sorted into chloroplasts in the respective transgenic plants (Figure 5B). This further confirmed that, AOX1a, the major AOX in plant cells, is also dually targeted to mitochondria and chloroplasts.

The immunoblotting assays showed AOX1a is a mitochondria and chloroplasts dually targeted protein, as AOX1b and AOX2. However, overexpressing W-AOX1b and W-AOX2 in *im* plants fully rescued the variegation phenotype of the mutant whereas transgenic *im* plants overexpressing W-AOX1a were as variegated as *im* (Figure 3). This phenotypic difference of transgenic plants carrying W-AOX1a, W-AOX1b, and W-AOX2 may be caused by the difference of PQH_2_ oxidase activity. When targeted into chloroplasts, C-mAOX1a is only able to partially rescue PTOX function in *im* plants, in contrast to C-mAOX1b and C-mAOX2 that fully rescue the *im* variegation phenotype (Figure 1), indicating that the PQH_2_ oxidase activity of AOX1a may be very low. Therefore, although a small amount of AOX1a can enter chloroplasts, the resulting PQH_2_ oxidase activity in chloroplasts is very low and not sufficient to rescue the variegation phenotype of *im*.

### Targeting Peptides of AOX1a, AOX1b, and AOX2 Can Direct the Mature Form of PTOX Into Chloroplasts

The immunoblotting assays, but not the fluorescence analysis of GFP-tagged proteins, showed that W-AOX1a is a dually targeted protein in transgenic *im* plants overexpressing W-AOX1a (Figures 4, 5). The inconsistency of these two analyses could be explained by that the low amount of AOX1a present in chloroplasts cannot be detected by the fluorescence analysis. Another possibility is that some AOX proteins are mis-targeted when overexpressed. Therefore, we think a third assay is necessary to clarify whether W-AOX1a is a dually targeted protein to mitochondria and chloroplasts.

A previous study showed that PTOX accumulates in large excess and that a trace amount of PTOX is sufficient to sustain normal chloroplast development and function (Fu et al., 2009). Therefore, the *im* mutant could be a highly sensitive tool to test whether a plant protein can be targeted into chloroplasts by examining the effect of its targeting peptide fused with the mature form of PTOX in *im* plants. If a targeting peptide directs PTOX into chloroplasts, the transgenic plants should show a green phenotype and normal growth; otherwise, the transgenic *im* plants should remain as variegated as *im*. Notably, it was confirmed in a very recent report (Sharma et al., 2019).

**FIGURE 6:**
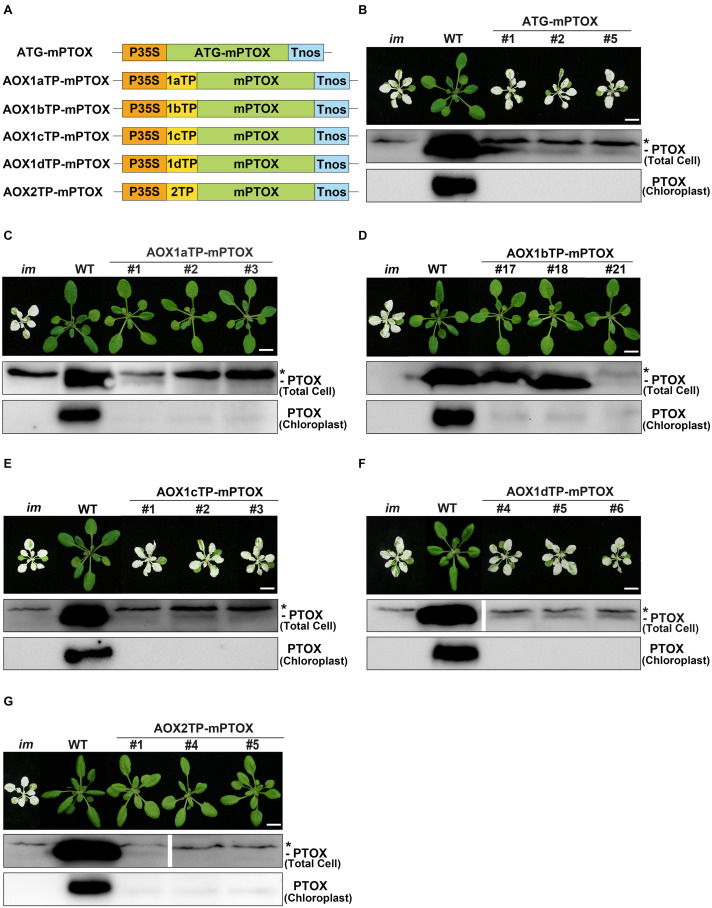
Expression of AOXTP-mPTOX constructs in *im*. **(A)** Schematic diagram of ATG-mPTOX, AOX1aTP-mPTOX, AOX1bTP-mPTOX, AOX1cTP-mPTOX, AOX1dTP-mPTOX, and AOX2TP-mPTOX constructs. P35S, Cauliflower Mosaic Virus 35S promoter; Tnos, terminator of nos (nopaline synthetase); ATG, initiation codon; 1aTP, AOX1a targeting sequence; 1bTP, AOX1b targeting sequence; 1cTP, AOX1c targeting sequence; 1dTP, AOX1d targeting sequence; 2TP, AOX2 targeting sequence. mPTOX, mature peptide sequence of PTOX. **(B–G)** Phenotypes of *im* mutant, WT, and three independent lines of transgenic *im* mutants carrying 35S-driven ATG-mPTOX, AOX1aTP-mPTOX, AOX1bTP-mPTOX, AOX1cTP-mPTOX, AOX1dTP-mPTOX, and AOX2TP-mPTOX. All plants were grown for 4 weeks with a photoperiod of 16 h light/8 h dark cycle under light (∼100 μmol⋅m^–2^s^–1^) after initial low light (∼20 μmol⋅m^–2^s^–1^) growth for 5 days as described in the “Materials and Methods” section. Total cell proteins were isolated from 10 mg fresh weight of leaf tissue (Total Cell). Chloroplast proteins were isolated from pellets obtained by centrifugation of lysed, Percoll gradient-purified chloroplasts (corresponding to 2 μg of chlorophyll). The protein samples were subjected to 12% SDS-PAGE and probed with PTOX antibody. Asterisk (*) marks unspecific bands detected by an antibody against PTOX. Bars = 1 cm.

At first, we transformed the mature form of PTOX lacking its CTP into *im*, and found that all transgenic plants showed the same variegation phenotype as *im*, indicating that chloroplast localization is required for the function of PTOX (Figures 6A,B). Then we generated constructs with the targeting peptides of AOXs fused with the mature PTOX and introduced these constructs into *im* (Figure 6). As expected, transgenic *im* plants carrying AOX1bTP-mPTOX or AOX2TP-mPTOX showed a normal green growth, and transgenic *im* plants carrying AOX1cTP-mPTOX or AOX1dTP-mPTOX showed the same variegation phenotype as *im*. These results showed that the targeting peptides of AOX1b and AOX2 target the mature PTOX into chloroplasts, while the targeting peptide of AOX1c or AOX1d failed to do so, implying that AOX1c and AOX1d are exclusively localized in mitochondria. Moreover, the AOX1aTP-mPTOX transgenic plants were green and grew normally as WT (Figure 6C) indicating that the targeting peptide of AOX1a can direct the mature PTOX into chloroplasts as well. To confirm these findings, we performed immunoblotting analyses on total cell protein samples and Percoll gradient-purified chloroplast samples from these transgenic plants with an antibody against PTOX. Trace amounts of PTOX were observed in the chloroplast samples from AOX1aTP-mPTOX, AOX1bTP-mPTOX, and AOX2TP-mPTOX transgenic lines, but no PTOX was detected in the chloroplast samples from ATG-mPTOX, AOX1cTP-mPTOX, or AOX1dTP-mPTOX transgenic lines (Figure 6). The phenotypic difference of transgenic *im* plants expressing W-AOX1a and AOX1aTP-mPTOX demonstrated again that the PQH_2_ oxidase activity of AOX1a may be very low.

In addition, we conducted a fluorescence analysis with the transient expression system, in which the above recombinant proteins were tagged with GFP at their C-termini and transiently expressed in *N. benthamiana*. The same results were observed as with the localization analysis of GFP-tagged W-AOXs in Figure 4. The fluorescence signals analysis showed that AOX1bTP-mPTOX and AOX2TP-mPTOX were dually targeted proteins present in mitochondria and chloroplasts; while both AOX1cTP-mPTOX and AOX1dTP-mPTOX were exclusively localized in mitochondria (Supplementary Figure 4). Fluorescence signals of the GFP-tagged AOX1aTP-mPTOX barely overlapped with the auto-fluorescence of chloroplasts (Supplementary Figure 4), consistent with the result of Figure 4B, suggesting that the amount of protein entering chloroplasts could be below the detecting limit of the fluorescence analysis.

### Overexpressed W-AOX1b and W-AOX2 Function Efficiently in Chloroplasts, but Differ From PTOX

**FIGURE 7:**
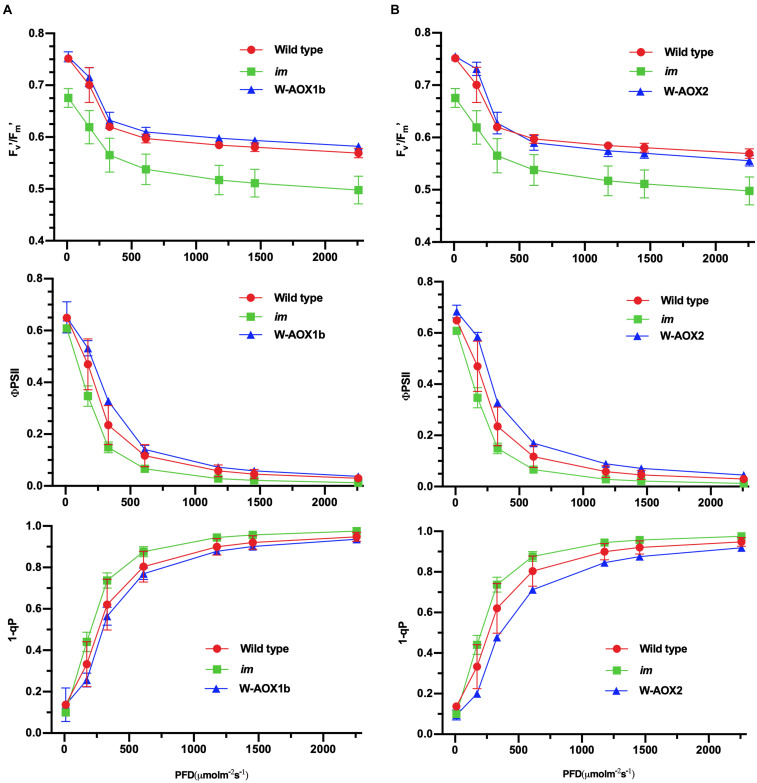
Chlorophyll *a* fluorescence measurements. Chlorophyll fluorescence parameters were measured on intact leaves from W-AOX1b **(A)** and W-AOX2 **(B)** and wild-type and *im* grown for 7 weeks in soil under the conditions as described in the “Materials and Methods” section. The parameters included the following: Fv’/Fm’, the maximum efficiency of PSII photochemistry under different photo flux densities (PFD); ΦPSII, the quantum efficiency of PSII photochemistry at different photo flux densities; 1-qP, the redox state of the Q_A_ electron acceptor of PSII (Maxwell and Johnson, 2000; Müller et al., 2001).

Transgenic *im* plants overexpressing W-AOX1b and W-AOX2 showed a green leaf appearance, but they differed from WT with longer and thinner leaves (Figure 3). We suspected that AOX1b and AOX2 cannot completely replace PTOX in chloroplasts, considering that their PQH_2_ oxidase activities are lower than PTOX. We conducted *in vivo* chlorophyll fluorescence analysis to determine whether the photosynthetic capacity is compromised in transgenic *im* plants overexpressing W-AOX1b and W-AOX2. The F_v_’/F_m_’, which represents the maximum efficiency of PSII photochemistry under different photon flux densities, was remarkably reduced in *im* (Figure 7). The F_v_’/F_m_’ values of W-AOX1b and W-AOX2 transgenic plants were similar as for WT indicating that the impaired PSII in *im* was restored by W-AOX1b and W-AOX2. ΦPSII, the quantum efficiency of PSII photochemistry at different photon flux densities (Maxwell and Johnson, 2000), in both W-AOX1b and W-AOX2 transgenic plants were slightly higher than for WT and *im* (Figure 7). The parameter 1-qP, which reflects the redox state of the Q_A_ electron acceptor of PSII, was lower in WT and transgenic plants than in *im* plants (Figure 7). This result indicated a more reduced PQ pool in *im*, whereas the PQ pool redox state in WT and transgenic plants is more balanced.

**FIGURE 8:**
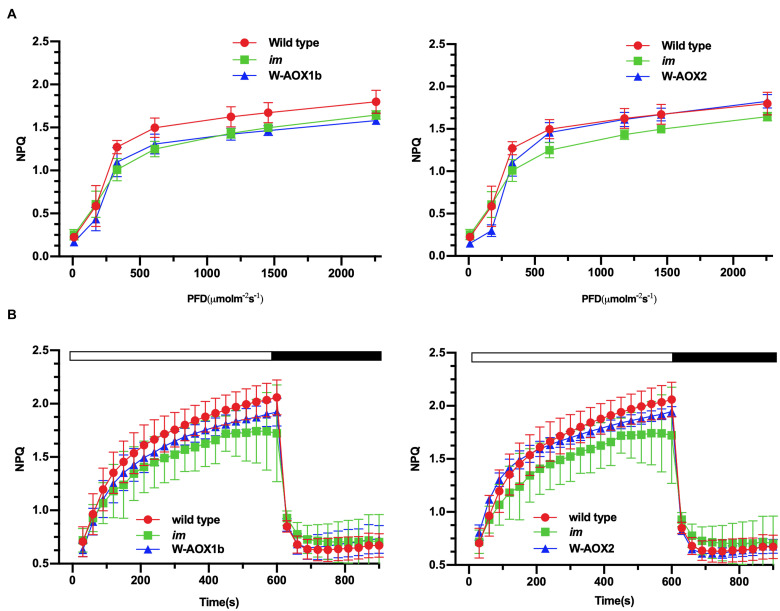
NPQ analyses. Steady state light response NPQ **(A)** and rapid induction and dark relaxation NPQ kinetics **(B)** were measured on intact leaves from wild-type, *im*, W-AOX1b, and W-AOX2 seedlings grown for 7 weeks in soil under low light (20 μmol⋅m^–2^s^–1^) for 5 days at 16-h light/8-h dark daylight cycle, and then were transferred to the normal growth condition (23°C, 16-h light/8-h dark daylight cycle, 100 μmol⋅m^–2^s^–1^) as described in the “Materials and Methods” section. The data represent the average ± SD of four independent experiments.

Non-photochemical quenching (NPQ), a measure of the ability of plants to dissipate excess light energy as heat (Müller et al., 2001), was found to be similar in *im* and W-AOX1b transgenic plants but lower than in WT (Figure 8A, left). In W-AOX2 transgenic plants, NPQ was close to that of *im* under light intensities less than 600 μmol m^–2^s^–1^, but similar to WT under high light intensities (≥600 μmol m^–2^s^–1^) (Figure 8A, right). This indicated that with increasing light intensity, AOX2 could dissipate excess light energy as heat more efficiently than AOX1b. The rapid induction of NPQ and the dark relaxation kinetics revealed that total NPQ is slightly higher in WT and transgenic plants than in *im* during the induction phase (Figure 8B). Taken together, most parameters were similar in the transgenic and WT plants, suggesting that steady state photosynthesis was not seriously perturbed by the presence of AOX1b and AOX2, but there were subtle differences between WT and these transgenic plants.

To investigate whether PSII assembly is affected in W-AOX1b and W-AOX2 transgenic plants, we examined the accumulation of photosynthetic complexes in thylakoid membranes by BN-PAGE. The results showed that the levels of the PSII supercomplexes (SCs) were almost the same in green tissues of *im*, WT, and both transgenic plants (Supplementary Figure 5).

## Discussion and Conclusion

Alternative oxidase is a terminal oxidase residing in the mitochondrial inner membrane to govern the balance of carbon and energy metabolism in mitochondria. Similarly, PTOX is a plastid terminal oxidase in the thylakoid membrane to maintain the PQ pool redox balance in chloroplasts (Wang and Fu, 2016). Here, taking advantage of Arabidopsis PTOX deficient mutant, we examined the functional relevance of AOXs and PTOX.

### AOXs Can Function as PQH_2_ Oxidases in Chloroplasts

Chloroplast PTOX and mitochondrial AOXs originated from an ancient di-iron oxidase and diverged early during evolution even before the endosymbiotic events giving rise to mitochondria and chloroplasts (McDonald and Vanlerberghe, 2006; Nobre et al., 2016). In agreement with their long divergent history, Arabidopsis PTOX and AOXs share a very low sequence identity, about 26% (Supplementary Figure 1). In structural aspect, PTOX and AOXs have their own unique features, for instance, the dimerization domain at the N-termini of AOXs and the extra sequence at the C-terminus of PTOX (Supplementary Figure 1; Fu et al., 2005, 2009). Given the structural difference and the different substrates available in their subcellular compartment, AOXs and PTOX have developed their substrate specificities for UQH_2_ and PQH_2_, respectively. Indeed, *in vitro* enzyme assays showed that PTOX specifically uses PQH_2_ as substrate and AOX only uses UQH_2_ (Josse et al., 2003; Fu et al., 2012; Yu et al., 2014).

However, targeting AOX1b, AOX1c, AOX1d, and AOX2 to chloroplasts rescued the abnormal phenotype of *im*, whereas targeting AOX1a to chloroplasts partially attenuated the leaf variegation of the mutant (Figure 1), demonstrating that all five AOXs could act as PQH_2_ oxidases although to a different extent. The results from *in vitro* assays (Josse et al., 2003; Fu et al., 2012; Yu et al., 2014) led us to conclude that the PQH_2_ oxidase activity of AOXs is much lower than that of PTOX. The PQH_2_ oxidase activity of AOXs except AOX1a is sufficient to complement the PTOX deficiency in *im*, because only trace amounts of PTOX are normally required for normal function of chloroplasts in Arabidopsis (Fu et al., 2009). As the major AOX in plant mitochondria, AOX1a should have developed a high specificity for UQH_2_ and possess a low PQH_2_ oxidase activity compared to the other minor AOXs. This may explain why AOX1a targeted to chloroplasts only partially rescues the variegation of *im*. This result, together with the previous study (Fu et al., 2012), indicated that the plasticity of substrate preference of related enzymes in plants could be higher than expected.

Whether PTOX could act as a UQH_2_ oxidase still remains unclear. The lack of obvious growth defects of AOX deficient mutants in Arabidopsis (Zhang et al., 2010, 2016) makes it very difficult to test the UQH_2_ oxidase activity of PTOX by targeting PTOX to mitochondria of AOX deficient mutants. The *E. coli* heme-deficient mutant strain, FN102, does not grow aerobically because it lacks the UQH_2_ oxidase activity of the heme-containing cytochrome pathway. However, the FN102 strain expressing AOX from *Trypanosoma brucei* could grow normally indicating that AOX could maintain respiratory electron transfer in the absence of the main cytochrome pathway chain in *E. coli* (Nihei et al., 2003). Therefore, it might be a good option to examine the UQH_2_ oxidase activity of PTOX by expressing this protein in the *E. coli* FN102 strain.

### AOX1a, AOX1b, and AOX2 May Be Dually Targeted Proteins in Mitochondria and Chloroplasts

Traditionally, proteins were thought to be only targeted to a single specific subcellular location. Since the pea glutathione reductase was found to be present in both mitochondria and chloroplasts in transgenic tobacco (Creissen et al., 1995), hundreds of plant proteins were identified to be targeted to multiple locations within the cell (Carrie and Small, 2013; Carrie and Whelan, 2013; Baudisch et al., 2014; Sharma et al., 2018). Currently over 100 proteins are found in both mitochondria and chloroplasts which have attracted considerable interest for their shared functions between these two energy-converting organelles. So far, the majority of identified dually targeted proteins are involved in essential common processes in mitochondria and chloroplasts, such as DNA replication, transcription, translation, and protein homeostasis (Baudisch et al., 2014; Sharma et al., 2018). The dually targeted proteins were thought to contain an ambiguous dual targeting peptide (dTP) at their N-termini which could direct passenger proteins into both chloroplasts and mitochondria (Ge et al., 2014; Sharma et al., 2018). Mitochondrial specific targeting peptides harbor multiple arginine residues and a hydrophobic sequence motif in the N-terminal region. This unique feature was thought to convey mitochondrial specificity and avoidance of import into chloroplasts (Ge et al., 2014; Lee et al., 2019).

We observed fluorescence signals from GFP-tagged W-AOXs all overlaid well with the fluorescence signals of the mitochondrial specific dye, Mt-Mcherry (Figure 4A), indicating that all five AOXs are primarily located in mitochondria as expected. While a portion of signals from the GFP-tagged W-AOX1b and W-AOX2 overlapped well with auto-fluorescence of chloroplasts, this was not the case for GFP-tagged W-AOX1a (Figure 4). However, we did observe that overexpressed W-AOX1a, as well as W-AOX1b and W-AOX2, present in purified chloroplasts based on immunoblotting assays (Figure 5). To clarify the discrepancy of the results from the two different assays, we fused five AOX targeting peptides with the mature form of PTOX, and observed that the targeting peptide of AOX1a, as well as those of AOX1b and AOX2, could direct PTOX to enter chloroplasts (Figure 6C). Altogether, it demonstrated that AOX1a could also be targeted into chloroplasts although the amount was too small to be detected by the GFP fluorescence assay. It has been reported that some of the overexpressed proteins could be mis-targeted to wrong organelles (Lisenbee et al., 2003; Vitali et al., 2018; Zhang et al., 2019). However, we examined more than 50 independent lines of W-AOX1c and W-AOX1d transgenic *im* plants, and found that none of those lines are green. In addition, no green phenotype was found in independent lines of AOX1cTP-mPTOX and AOX1dTP-mPTOX transgenic *im* plants. All implied that the variegation suppression phenotype in transgenic lines were not likely caused by protein mis-targeting. Based on the current data and the previous results (Fu et al., 2012), we surmised that AOX1a, AOX1b, and AOX2 may be dually targeted proteins in mitochondria and chloroplasts, while AOX1c and AOX1d are specifically localized in mitochondria.

Alternative oxidase family members have been assumed to specifically reside in mitochondria (Clifton et al., 2006; Vanlerberghe, 2013). All five Arabidopsis AOXs are predicted to be localized in mitochondria by various subcellular targeting prediction programs (Supplementary Table 1). Analysis of AOX targeting peptides indicated that all contain multiple arginine residues and a hydrophobic sequence motif at the N-terminus (Supplementary Figure 3) fitting the profile of typical mitochondria-targeted proteins (Ge et al., 2014; Lee et al., 2019). Previously AOX1a was used as a specific mitochondrial marker to investigate whether a plant protein is targeted into mitochondria (Carrie et al., 2009).

However, the result that three AOXs (AOX1a, AOX1b, and AOX2) may be dually targeted proteins is not in agreement with the current knowledge of protein dual targeting into mitochondria and chloroplasts. Previously, a predicted specific mitochondrial protein, fumarate hydratase (FumH), was reported to be a dually targeted protein to mitochondria and chloroplasts (Baudisch et al., 2014). Together, these unexpected results suggested that the mechanisms of protein targeting into organelles are much more complex than expected, and dual targeting properties could be more abundant than anticipated.

### Could AOXs Enter Chloroplasts and Function as PQH_2_ Oxidase in Non-transgenic Plants?

AOX1a, AOX1b, and AOX2 may be dually targeted proteins to mitochondria and chloroplasts, and overexpression of AOX1b and AOX2 in *im* could rescue the mutant phenotype. However, the question arises whether AOX1a, AOX1b, and AOX2 could enter chloroplasts and function as PQH_2_ oxidase in non-transgenic plants?

Most AOX genes are expressed at low content in plants except for AOX1a which is the most dominantly expressed isoform (Supplementary Figure 2) and expressed in all plant tissues and organs (Clifton et al., 2006). AOX1b is predominantly expressed in pollen and stamen, and AOX2 expression appears to be specific to mature seed and young inflorescences (Nakabayashi et al., 2005; Clifton et al., 2006). The transcript level of AOX1a dramatically elevates in response to multiple stress conditions. In contrast, AOX2 transcription is suppressed under most stress conditions, but remarkably induced by treatments affecting chloroplast function such as application of paraquat, cysteine, and norflurazon, suggesting a potential role for AOX2 in inter-organellar signaling between chloroplasts and mitochondria (Clifton et al., 2005). It is possible that AOX1a and AOX2 could enter chloroplasts once their expression is induced to high levels, especially under conditions where the function of chloroplasts is impaired. AOX1b and AOX2 were poorly expressed in WT plants under normal conditions (Supplementary Figure 2). We therefore suspected that they are not required for maintaining general mitochondrial functions but could play important roles under stress conditions.

Mitochondrial AOX was reported to act as a buffer pool to consume the excessive reducing power in chloroplasts through the malate/oxaloacetate shuttle (Noguchi and Yoshida, 2008; Taniguchi and Miyake, 2012). However, we found that only chloroplast-localized AOXs are able to suppress *im* variegation (Figures 1, 3). Therefore, the effects on chloroplast redox poise mediated by mitochondria-localized AOX was not pursued in this study.

### The Arabidopsis *im* Mutant Can Be Used as a Tool to Examine Whether a Protein Can Be Targeted to Chloroplasts

Currently, several approaches have been established to determine the targeting specificity of proteins, including *in silico* targeting prediction, *in organelle* protein transport experiments, fluorescence analysis of GFP-tagged proteins, organelle fractionation with immunoblotting assays (Carrie and Small, 2013; Sharma et al., 2018).

The fluorescence analysis of GFP-tagged proteins is the most frequently used approach to determine the sub-cellular location of a protein. However, it is not sensitive enough to detect whether a minor part of an abundant protein is localized in different parts of the cell such as AOX1a (Figure 4). Organelle purification plus immunoblotting is a powerful method to determine whether a protein enters a specific organelle, but it relies on the quality of subcellular fractionation and on suitable antibodies against proteins of interest.

In this study, we fused targeting peptides of AOXs with the mature form of PTOX, and found that the targeting peptide of AOX1a, as well as those of AOX1b and AOX2, could evidently direct PTOX to chloroplasts (Figure 6C). Trace amounts of PTOX are sufficient to maintain normal chloroplast development and function (Fu et al., 2009) making the *im* mutant a sensitive tool to test whether a targeting peptide could direct mature PTOX into chloroplasts. This approach had also been validated in a very recent study (Sharma et al., 2019). The *im* mutant grows well under low light and can be easily transformed. The striking variegation phenotype of the mutant enables us to examine whether a targeting peptide functions as a chloroplast transit peptide based on the phenotype of transgenic T_1_ plants. If transgenic plants are green, it indicates that this is indeed the case whereas the variegation phenotype indicates that it is not (Figure 6).

## Data Availability Statement

The original contributions presented in the study are included in the article/Supplementary Material, further inquiries can be directed to the corresponding author/s.

## Author Contributions

AF and MX designed the research. DW, CW, CL, HS, JQ, HC, and WF performed the experiments. DW, YW, MX, FW, BL, YH, and AF analyzed the data. DW, MX, and AF wrote the manuscript with contributions and approval from all authors.

## Conflict of Interest

The authors declare that the research was conducted in the absence of any commercial or financial relationships that could be construed as a potential conflict of interest.

## Footnotes

1 https://www.arabidopsis.org
