# Supplementary material for: Functional Relationship of Arabidopsis AOXs and PTOX Revealed via Transgenic Analysis
Source: Front Plant Sci. 2021 Jul 2;12:692847. doi: 10.3389/fpls.2021.692847 (PMC8336870; doi:10.3389/fpls.2021.692847)
Supplement: Supplementary file 1 [file Data_Sheet_1.PDF]

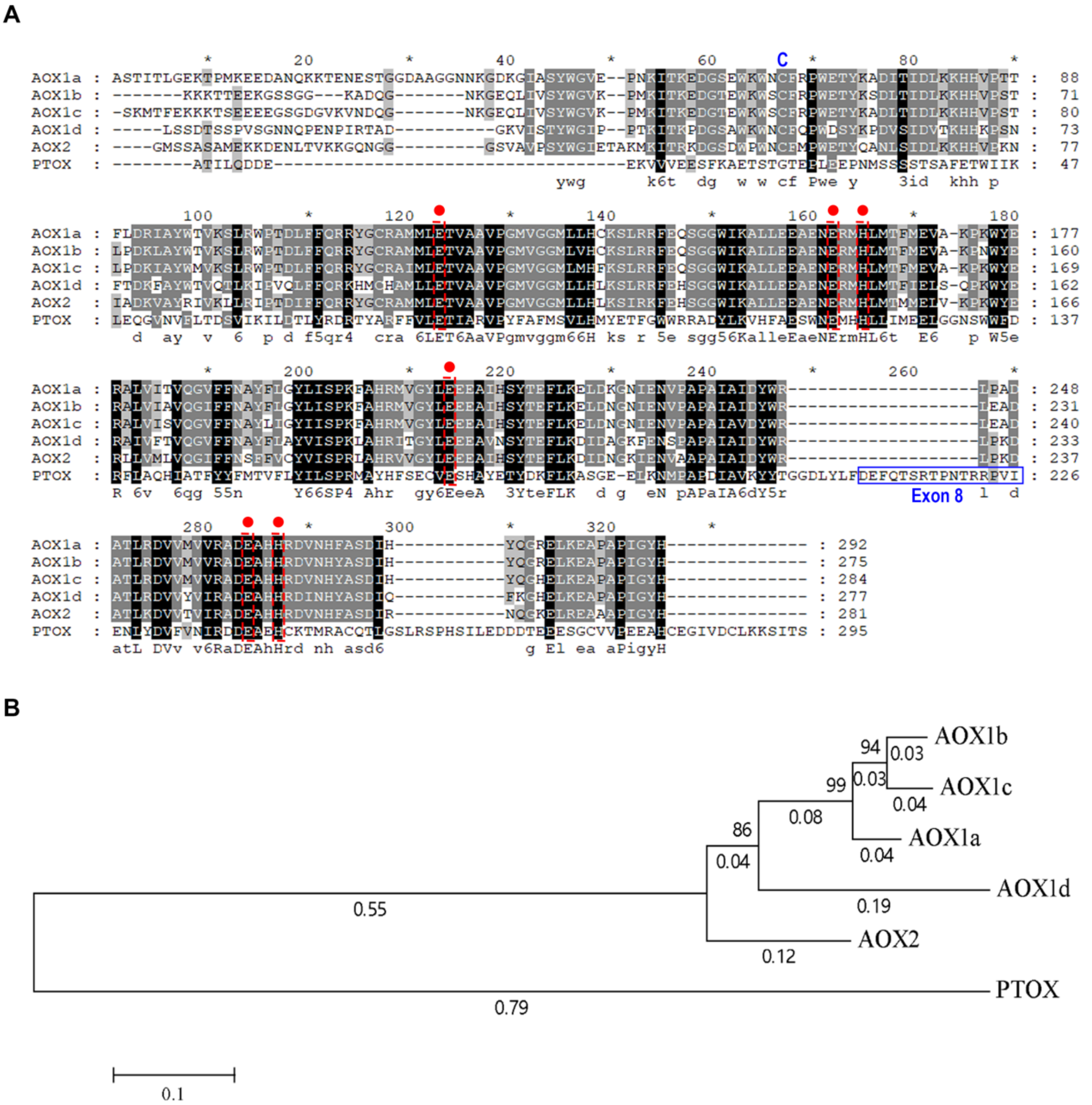

Supplementary Figure 1. Sequence comparison between Arabidopsis AOXs and PTOX.

(A) ClustalW multiple sequence alignment was performed using the protein mature sequences encoded by the five *AOX* genes and *PTOX* gene of Arabidopsis. Sequences highlighted in black represent completely conserved regions. The six iron binding sites are indicated by solid red circles. AOX has a unique dimerization domain (D domain) that contains a regulatory Cys which is indicated by a blue C. Whereas PTOX has a unique Exon 8 domain required for protein stability, which is indicated by a blue rectangle. **(B)** Phylogenetic Relationships of AOXs and PTOX in Arabidopsis. Phylogenies were generated using mature protein sequences of AOXs and PTOX. The phylogenetic tree was constructed with MEGA6 based on the multiple sequence alignment profile (Supplementary Figure 1A) using the Neighbor-Joining method (Saitou and Nei, 1987) and a 1,000 bootstrap resampling value (Felsenstein, 1985).

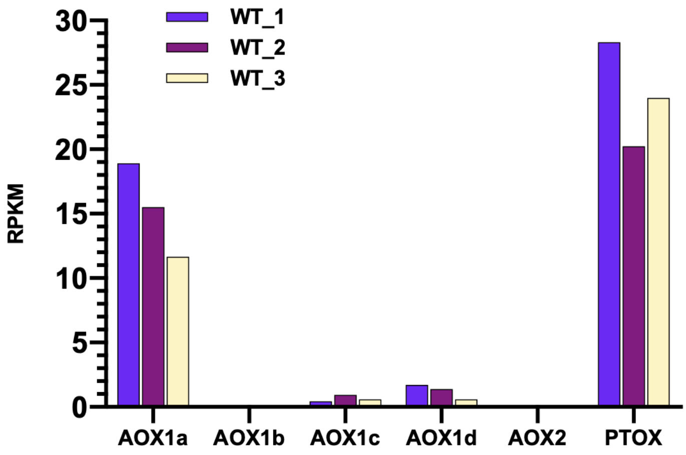

Supplementary Figure 2. Expression of *AOXs* and *PTOX* in wild type.

Expression profiles of five *AOX* genes, and *PTOX* gene in wild type Arabidopsis were obtained from RNA-seq data. RPKM (reads per kilobase of transcript per million reads mapped) was used to measure gene or transcript expression levels (Zhao et al., 2020).

AOX1a(one R,fifteen hydrophobic amino acids)

MMITRGGAKAAKSLLVAAGPRLFSTVRTVSSHEALSASHILKPGVTSAWIWTRAPTIGGMRF

AOX1b(two R,nine hydrophobic amino acids)

MMMSRRYGAKLMETAVTHSHLLNPRVPLVTENIRVPAMGVVRVFSKMTFE

AOX1c(two R,nine hydrophobic amino acids)

MITTLLRRSLLDASKQATSINGILFHQLAPAKYFRVPAVGGLRDF

AOX1d(three R,seven hydrophobic amino acids)

MSYRSIYRTLRPVLSSSVQSSGLGIGGFRGHLISHLPNVRL

AOX2(two R,ten hydrophobic amino acids)

MSQLITKAALRVLLVCGRGNCNMFVSSVSSTSVMKSPYEITAPMRIHDWCGGFGDFKIGSKHVQGNFNLRWM

PTOX(0 R,six hydrophobic amino acids)

MAAISGISSGTLTISRPLVTLRRSRAAVSYSSSHRLLHHLPLSSRRLLLRNNHRVQ

Supplementary Figure 3. Analysis of targeting peptides of AOXs and PTOX.

Multiple Arginine residues (solid black circles) and hydrophobic residues (solid red triangle) were marked as described previously (Lee et al., 2019).

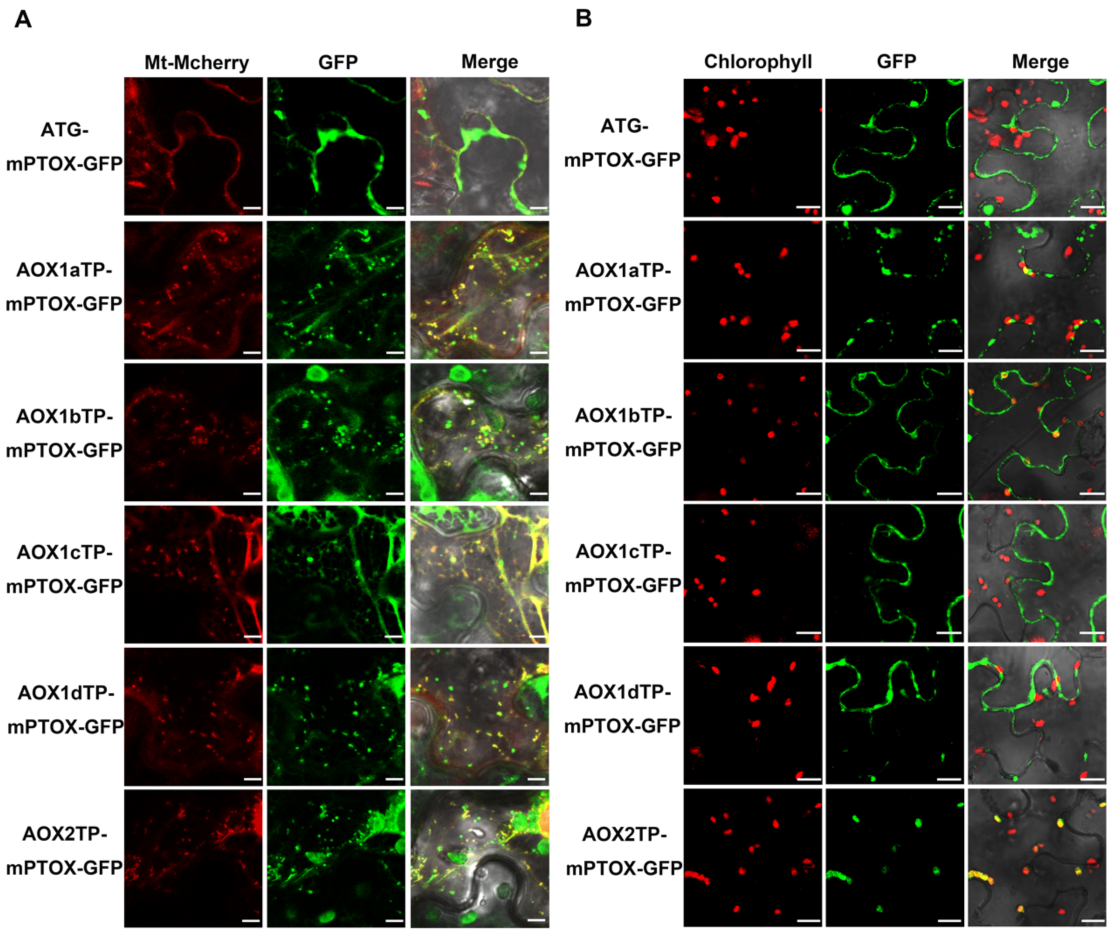

Supplementary Figure 4. The fluorescence analysis of GFP-tagged AOX1aTP-mPTOX, AOX1bTP-mPTOX, AOX1cTP-mPTOX, AOX1dTP-mPTOX, and AOX2TP-mPTOX.

ATG-mPTOX, AOX1aTP-mPTOX, AOX1bTP-mPTOX, AOX1cTP-mPTOX, AOX1dTP-mPTOX, and AOX2TP-mPTOX tagged with a C-terminal GFP were transiently expressed under the control of 35S promoter in *N. benthamiana* leaves respectively and observed by confocal microscopy. **(A)** In each case, images of mitochondrial Mcherry fluorescence (Mt-Mcherry), GFP fluorescence (GFP), and merged Mcherry and GFP fluorescence with bright-field (Merge) are shown. Scale bar = 5 µm. **(B)** In each case, images of chlorophyll autofluorescence (Chl), GFP fluorescence (GFP), and merged chlorophyll and GFP fluorescence with bright-field (Merge) are shown. Scale bar = 20 µm.

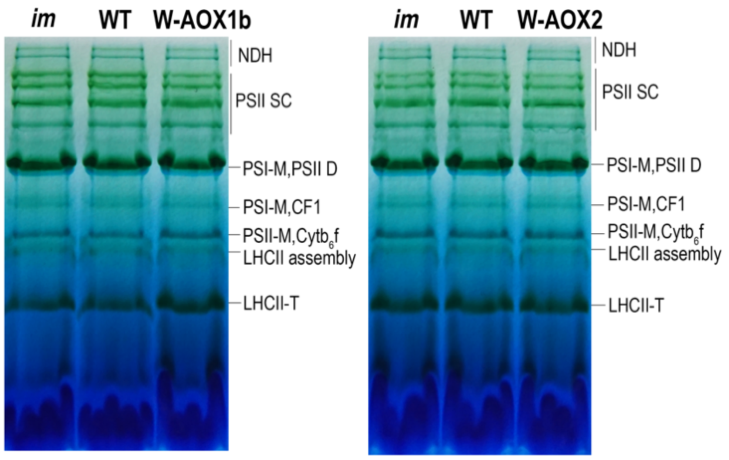

Supplementary Figure 5. BN gel of thylakoid membrane protein complexes from *im*, WT, W-AOX1b and W-AOX2.

Thylakoid membranes solubilized by 1% β-DM were separated by BN-PAGE (20 μg chlorophyll per lane). NDH, NAD(P)H dehydrogenase; PSII SC, PSII supercomplex; PSI-M, PSI monomer; PSII-D, PSII dimer; Cytb$_6$f, cytochrome b$_6$f; LHCII-T, PSII light-harvesting complex trimer; LHCII monomer, PSII light-harvesting complex monomer.

Supplementary Table 1 Subcellular location of AOXs and PTOX predicted by various programs.

Protein	Predotar			Target P 1.1					Plant-mPLoc	Euk-mPLoc 2.0
	cTP	mTP	Loc.	cTP	mTP	sp	Other	Loc.	Loc.	Loc.
AOX1a	0.00	0.58	M	0.313	0.612	0.016	0.016	M	M	M
AOX1b	0.00	0.41	M	0.062	0.640	0.097	0.132	M	M	M
AOX1c	0.01	0.83	M	0.044	0.667	0.085	0.304	M	M	M
AOX1d	0.00	0.74	M	0.139	0.886	0.009	0.022	M	M	M
AOX2	0.01	0.56	M	0.128	0.512	0.314	0.017	M	M	M
PTOX	0.96	0.36	C	0.965	0.350	0.002	0.011	C	C	C

This table shows the performance of these prediction tools on proteins of AOXs and PTOX. TargetP (http://www.cbs.dtu.dk/services/TargetP-1.1/index.php), Predotar (http://www.inra.fr/Internet/Prod-uits/Predotar/), Plant-mPLoc (http://www.csbio.sjtu.edu.cn/bioinf/plant-multi/), Euk-mPLoc2.0 (http://www.csbio.sjtu.edu.cn/bioinf/euk-multi-2/). M stands for mitochondrial, C for chloroplast. chloroplast transit peptide (cTP), mitochondrial targeting peptide (mTP) or secretory pathway signal peptide (SP). The column Loc. indicates the predicted location.

Supplementary Table 2 List of primers used in this study.

**Gene**	**Primer name**	**Sequences (5'->3')**
AOX1a	AOX1aF	CCCCGAATTCATGATGATAACTCGCGGTGGAGCC
AOX1a	AOX1aR	CCCCGTCGACTCAATGATACCCAATTGGAGCTGG
AOX1b	AOX1bF	CCCCGAATTCATGATGATGAGTCGTCGCTATGG
AOX1b	AOX1bR	CCCCGTCGACTCAATGATATCCAATGGGAGCTGG
AOX1c	AOX1cF	CCCCGTCGACATGATCACTACATTACTCCGTCG
AOX1c	AOX1cR	CCCCCTCGAGTCAGTGATATCCTATAGGAGCTG
AOX1d	AOX1dF	CCCCCCGGATCCATGTCCTACAGATCGATTTACCG
AOX1d	AOX1dR	CCCCGTCGACTTAATGATATCCAATAGGAGCCG
AOX2	AOX2F	CCCCGGATCCATGAGTCAACTCATTACGAAAGC
AOX2	AOX2R	CCCCGTCGACTTAGTGATAACCAATCGGAGCTG
AOX1a	AOX1aSF	CCTCGAATTCGCTAGCACGATCACTCTGGGAGAG
AOX1a	AOX1aSR	CCTCCTCGAGCGCGATTCCTTTATCTCCCTTG
AOX1b	AOX1bSF	CCTCGAATTCGCCAAGCTAATGGAAACTGCTGTG

AOX1b	AOX1bSR	CCTCCTCGAGGAAAACTCTCACGACTCCCATCGC
AOX1c	AOX1CSF	CCTCGAATTCAGCAAGATGACATTTGAAAAGAAG
AOX1c	AOX1CSR	CCTCCTCGAGAACTATTAATTGTTCCCCTTTGTTACC
AOX1d	AOX1DSF	CCTCGAATTCTTGAGCTCTGACACGTCATCTCC
AOX1d	AOX1DSR	CCTCCTCGAGAGGTATACCCCAATAAGTGGATATAA
AOX2	AOX2SF	CCTCGAATTCGGTATGAGTTCTGCATCGGCGATG
AOX2	AOX2SR	CCTCCTCGAGTGCTGTCTCTATTCCCCAATAAC
AOX1a	AOX1A_F1	CGCCTCTAGAATGATGATAACTCGCGGTGGAGCC
AOX1a	AOX1A_R1	CGCCCCGGATCCTCAATGATACCCAATTGGAGCTGG
AOX1a	AOX1A_GR1	CGCCCCGGATCCATGATACCCAATTGGAGCTGGAGC
AOX1b	AOX1B_deF1	GCCATGGGAGACATATAAATC
AOX1b	AOX1B_deR1	CCTAAAGCAACTCCATTTCC
AOX1b	AOX1B_deF2	CAGAGGCGGTACGGATGCAG
AOX1b	AOX1B_deR2	CTGGAAGAAAAGATCGGTAGGC
AOX1b	AOX1B_deF3	GATATTCATTACCAAGGTCGTG
AOX1b	AOX1B_deR3	CGGATGCATAGTGGTTAACATC
AOX1b	AOX1b_deGF	CCATGGGAGACATATAAATCAGATC
AOX1b	AOX1b_deGR	CCTAAAGCAACTCCATTTCCATTCAG
AOX1b	1bCDS_F(XbaI)	CCGCTCTAGAATGATGATGAGTCGTCGCTATGGAG
AOX1b	1bCDS_R(BamHI)	CGCCCCGGATCCTCAATGATATCCAATGGGAGCTGG
AOX1b	1bCDS_GR(BamHI)	CGCCCCGGATCCATGATATCCAATGGGAGCTGGAGC
GFP	GFP_F（BamHI）	CGCCCCGGATCCATGGTGAGCAAGGGCGAGGAGCT
GFP	GFP_R（BamHI）	CGCCCCGGATCCTTACTTGTACAGCTCGTCCATGC
GFP	GFP(BglII)	CGCCAGATCTTTACTTGTACAGCTCGTCCATGC
AOX1c	AOX1C_F1	CCCCTCTAGAATGATCACTACATTACTCCGTCG
AOX1c	AOX1C_R0	CGCCAGATCTTCAGTGATATCCTATAGGAGCTG
AOX1c	AOX1C_GR0	CGCCAGATCTGTGATATCCTATAGGAGCTGGAG
AOX1d	AOX1D_F1	CCCCTCTAGAATGTCCTACAGATCGATTTACCG
AOX1d	AOX1D_R1	CCCCCCGGATCCTTAATGATATCCAATAGGAGCCG
AOX1d	AOX1D_GR1	CCCCCCGGATCCATGATATCCAATAGGAGCCGGAG
AOX2	AOX2_F1	CCCCTCTAGAATGAGTCAACTCATTACGAAAGC
AOX2	AOX2_R1	CGCCCCGGATCCTTAGTGATAACCAATCGGAGCTG
AOX2	AOX2_GR1	CGCCCCGGATCCGTGATAACCAATCGGAGCTGCTGC
CTP-AOX1a	pB003-CTP-AOX1aF	CGCCTCTAGAATGGCTTCCTCTATGCTCTCTTCC
CTP-AOX1a	pB003-CTP-AOX1aR	CGCCCCGGATCCTCAATGATACCCAATTGGAGCTGG
CTP-AOX1b	CTP-AOX1BF	CGCGGATCCGCCAAGCTAATGGAAACTGCTGTGA
CTP-AOX1b	CTP-AOX1BR	CGCGGATCCTCAATGATATCCAATGGGAGCTGGAG
CTP-AOX1c	CTP-AOX1CF2	ATAGGCCCGGGAAGCAAGATGACATTTGAAAAGAA
CTP-AOX1c	CTP-AOX1CR1	ATACGCCCGGGTCAGTGATATCCTATAGGAGCTGG
CTP-AOX1d	CTP-AOX1DF	CGCGGATCCTTGAGCTCTGACACGTCATCTCC
CTP-AOX1d	CTP-AOX1DR	CGCGGATCCTTAATGATATCCAATAGGAGCCGG

CTP-AOX2	CTP-AOX2F	CGCGGATCCGGTATGAGTTCTGCATCGGCGATG
CTP-AOX2	CTP-AOX2R	CGCGGATCCTTAGTGATAACCAATCGGAGCTGC
CTP-AOX1a	CTP_spyF(SpeI)	CCGCACTAGTATGGCTTCCTCTATGCTCTCTTCCG
CTP-AOX1a	AOX1a_spyR1(XhoI)	CCGCCTCGAGATGATACCCAATTGGAGCTGGAG
CTP-AOX1b	AOX1B_spyR1(XhoI)	CCGCCTCGAGATGATATCCAATGGGAGCTGGAGCT
CTP-AOX1c	AOX1C_spyR1(XhoI)	CCGCCTCGAGGTGATATCCTATAGGAGCTGGAGC
CTP-APX1d	AOX1d_spyR1(XhoI)	CCGCCTCGAGATGATATCCAATAGGAGCCGGAGC
CTP-AOX2	AOX2_spyR1(XhoI)	CCGCCTCGAGGTGATAACCAATCGGAGCTGCTGCT
ATG-PTOXmp	pB003-ATGPTOXmpF	CCGCTCTAGAATGGCAACGATTTTGCAAGACGATG
ATG-PTOXmp	pB003-ATGPTOXmpR	CCGCCCGGATCCTTAACTTGTAATGGATTTCTTGAG
AOX1aTP	AOX1aTP_F(XbaI)	CCGCTCTAGAATGATGATAACTCGCGGTGGAGCC
AOX1aTP	AOX1aTP_R1	GAATCTCATACCTCCAATCGTCGGAG
PTOXmp	PTOXmp_F	GCAACGATTTTGCAAGACGATGAAGAGAAAG
PTOXmp	PTOXmp_R(BamHI)	CGCCGGATCCTTAACTTGTAATGGATTTCTTGAGGC
AOX1bTP	pB003AOX1bTP_F	CCGCTCTAGAATGATGATGAGTCGTCGCTATGGAG
AOX1bTP	AOX1bTP_R	CTCAAATGTCATCTTGCTGAAAACT
AOX1cTP	AOX1cTP_F（XbaI）	CCGCTCTAGAATGATCACTACATTACTCCGTCGCTC
AOX1cTP	AOX1cTP_R1	GAAATCTCTCAGACCTCCCACTGCC
AOX1dTP	AOX1dTP_F(XbaI)	CCGCTCTAGAATGTCCTACAGATCGATTTACCGC
AOX1dTP	AOX1dTP_R	TAGCCGAACATTGGGCAAGTGGCTAA
AOX2TP	AOX2TPF_XbaI	CCGCTCTAGAATGAGTCAACTCATTACGAAAGCAGC
AOX2TP	AOX2TPR	CATCCACCTCAAGTTAAAATTTCC
ATG-PTOXmp	ATGPTOX_F(pCAM)	CCGCAGATCTATGGCAACGATTTTGCAAGACGATG
ATG-PTOXmp	PTOX_R(pCAM)	CCGCAGATCTACTTGTAATGGATTTCTTGAGGC
AOX1aTP-PTOXmp	AOX1aTP_F(pCAM)	CCGCAGATCTATGATGATAACTCGCGGTGGAGC
AOX1bTP-PTOXmp	AOX1bTP_F(pCAM)	CCGCAGATCTATGATGATGAGTCGTCGCTATGGAG
AOX1cTP-PTOXmp	AOX1cTP_F(pCAM)	CCGCAGATCTATGATCACTACATTACTCCGTCG
AOX1dTP-PTOXmp	AOX1dTP_F(pCAM)	CCGCAGATCTATGTCCTACAGATCGATTTACCG
AOX2TP-PTOXmp	AOX2TP_F(pCAM)	CCGCAGATCTATGAGTCAACTCATTACGAAAGC
